# Immunotoxin-Induced Ablation of the Intrinsically Photosensitive Retinal Ganglion Cells in Rhesus Monkeys

**DOI:** 10.3389/fneur.2018.01000

**Published:** 2018-11-27

**Authors:** Lisa A. Ostrin, Christianne E. Strang, Kevin Chang, Ashutosh Jnawali, Li-Fang Hung, Baskar Arumugam, Laura J. Frishman, Earl L. Smith, Paul D. Gamlin

**Affiliations:** ^1^College of Optometry, University of Houston, Houston, TX, United States; ^2^Department of Psychology, University of Alabama at Birmingham, Birmingham, AL, United States; ^3^Department of Ophthalmology and Visual Sciences, University of Alabama at Birmingham, Birmingham, AL, United States

**Keywords:** melanopsin, ipRGCs, intrinsically photosensitive retinal ganglion cells, immunotoxin, pupil, rhesus monkey

## Abstract

**Purpose:** Intrinsically photosensitive retinal ganglion cells (ipRGCs) contain the photopigment melanopsin, and are primarily involved in non-image forming functions, such as the pupillary light reflex and circadian rhythm entrainment. The goal of this study was to develop and validate a targeted ipRGC immunotoxin to ultimately examine the role of ipRGCs in macaque monkeys.

**Methods:** An immunotoxin for the macaque melanopsin gene (*OPN4*), consisting of a saporin-conjugated antibody directed at the N-terminus, was prepared in solutions of 0.316, 1, 3.16, 10, and 50 μg in vehicle, and delivered intravitreally to the right eye of six rhesus monkeys, respectively. Left eyes were injected with vehicle only. The pupillary light reflex (PLR), the ipRGC-driven post illumination pupil response (PIPR), and electroretinograms (ERGs) were recorded before and after injection. For pupil measurements, 1 and 5 s pulses of light were presented to the dilated right eye while the left pupil was imaged. Stimulation included 651 nm (133 cd/m^2^), and 4 intensities of 456 nm (16–500 cd/m^2^) light. Maximum pupil constriction and the 6 s PIPR were calculated. Retinal imaging was performed with optical coherence tomography (OCT), and eyes underwent OPN4 immunohistochemistry to evaluate immunotoxin specificity and ipRGC loss.

**Results:** Before injection, animals showed robust pupil responses to 1 and 5 s blue light. After injection, baseline pupil size increased 12 ± 17%, maximum pupil constriction decreased, and the PIPR, a marker of ipRGC activity, was eliminated in all but the lowest immunotoxin concentration. For the highest concentrations, some inflammation and structural changes were observed with OCT, while eyes injected with lower concentrations appeared normal. ERG responses showed better preserved retinal function with lower concentrations. Immunohistochemistry showed 80–100% ipRGC elimination with the higher doses being more effective; however this could be partly due to inflammation that occurred at the higher concentrations.

**Conclusion:** Findings demonstrated that the *OPN4* macaque immunotoxin was specific for ipRGCs, and induced a graded reduction in the PLR, as well as, in ipRGC-driven pupil response with concentration. Further investigation of the effects of ipRGC ablation on ocular and systemic circadian rhythms and the pupil in rhesus monkeys will provide a better understanding of the role of ipRGCs in primates.

## Introduction

The intrinsically photosensitive retinal ganglion cells (ipRGCs) are located in the inner retina and express the photopigment melanopsin. The ipRGCs are involved in non-image forming functions, including photoentrainment of circadian rhythm and pupil size control (1, 2). Studies show that the ipRGCs also play a role in image formation, contributing to visual detection and temporal and color processing (3–5). They represent ~0.2–2% of the ganglion cell population, depending on species (6–9), and are characterized by large soma and broad dendritic fields (10). Multiple subtypes of ipRGCs have been identified (8, 11, 12), each demonstrating distinct molecular, morphological and functional characteristics. Five unique subtypes of ipRGCs have been identified in rodents (12), and two subtypes have been identified in primates (8). Axons run in the retinohypothalamic tract, and central projections include the suprachiasmatic nucleus, intergeniculate leaflet, olivary pretectal nucleus, and multiple other nuclei of the midbrain (6). With the recent characterization of the ipRGCs over the last 15–20 years, the full scope of ipRGCs in non-image and image forming processes in primates has yet to be fully elucidated.

The ipRGCs are stimulated intrinsically by light through activation of melanopsin (Opn4), with a peak sensitivity to short wavelength light at ~480 nm (4). Additionally, ipRGCs receive extrinsic input from the rod/cone pathway through contacts with cone bipolar cells and amacrine cells (8, 13). In macaques, the two subtypes of melanopsin cells depolarize in response to light in photopic conditions, and one subtype also responds to dim stimuli (4). Responses mediated by melanopsin exhibit slow kinetics, with activity persisting after stimulus offset (1). During the pupillary light reflex, initial pupil constriction is primarily attributed to rod and cone pathways, whereas maintained pupilloconstriction is primarily attributed to intrinsic ipRGC activity. The temporal properties of melanopsin activation contribute to sustained pupil constriction observed *in vivo* after light offset. Additionally, tonic pupil constriction in bright light is also attributed to melanopsin-driven pathways, as ganglion cells driven by cone input demonstrate light adaptation and rapid desensitization (14, 15).

Studies show that mice lacking rod and cone photoreceptors exhibit relatively normal circadian rhythm entrainment and pupil constriction to illumination (16, 17). Mice lacking melanopsin through gene deletion demonstrate diminished pupillary light reflexes at high irradiances (18) and attenuation in light-induced resetting of the circadian oscillator (19). Ruby et al. found that entrainment to the light/dark cycle and phase shifting were 40% lower in melanopsin knockout mice compared to wild-type mice (20). Mice lacking both rod and cone photoreceptors, as well as, ipRGCs show no pupillary responses (21).

The melanopsin photopigment is localized to ipRGCs, and has a highly unique amino acid sequence, making it ideal for lesioning studies. Previous studies have utilized saporin-conjugated immunotoxins for targeted ablation of the melanopsin containing ipRGCs in mice (22) and rats (23). Ingham et al. showed that the immunotoxin rapidly and permanently ablated ~70% of the ipRGC population in rats, with no alterations in non-melanopsin-containing retinal cells (23). Mice lacking melanopsin cells showed attenuation in circadian photosensitivity and decreased light-induced negative masking. Specifically, mice demonstrated an impaired ability to entrain to photoperiod, suggesting that the experimental animals were less sensitive to light.

The development of an effective immunotoxin for the primate melanopsin containing cells is an important step in elucidating the roles of ipRGCs in non-image forming and image forming functions. The goal of this study was to develop and validate a targeted ipRGC immunotoxin to ultimately examine the role of these cells in primates.

## Materials and methods

Subjects were six rhesus monkeys (*Macaca mulatta*), ages 1–4 years (Table 1), that were reared under fluorescent ambient lighting on a 12 h light/12 h dark cycle [for husbandry details see (24)]. Procedures were approved by the Institutional Animal Care and Use Committee at the University of Houston and conformed to the ARVO statement for the Use of Animals in Ophthalmic and Vision Research.

**Table 1:** Age, sex, and immunotoxin dose for the experimental animals, *Macaca mulatta*.

**Subject ID**	**Age (years)**	**Sex**	**Dose (μg)**
550	2.6	Female	50
552	2.6	Male	10
520	4	Male	10
526	4	Male	3
527	4	Male	1
609	1	Female	0.316

To develop the primate OPN4 immunotoxin, an affinity-purified rabbit polyclonal antibody directed against the N-terminus extracellular domain of OPN4 was generated. Specifically, a peptide consisting of the 19 amino acid residues from the N-terminus of hOPN4 (MNPPSGPRVPPSPTQEPSC) was synthesized and conjugated to KLH (Genscript, Piscataway, NJ). This 19 amino acid sequence is common to humans and macaque monkeys. The conjugate was used to immunize two rabbits, and the resulting antisera was affinity-purified to a concentration of 1.545 mg/ml (Genscript). Custom conjugation by Advanced Targeting Systems (San Diego, CA) generated a saporin-conjugated anti-h-OPN4 antibody at a concentration of 1.1 mg/ml.

The saporin-conjugated anti-hOPN4 antibody solution was diluted in a vehicle of sterile balanced salt solution (BSS) for intravitreal injection to deliver 50 μg (*n* = 1, animal 550), 10 μg (*n* = 2, animals 552 and 520), 3.16 μg (*n* = 1, animal 526), 1 μg (*n* = 1, animal 527), or 0.316 μg (*n* = 1, animal 609) to the right eyes of six animals. The vehicle alone was injected in the left eyes. For injections, animals were anesthetized with an intramuscular injection of 30 mg/kg ketamine and 3 mg/kg acepromazine. The eye adnexa was washed with betadine, topical proparacaine was instilled, and the eye was rotated to inject through a pars plana approach. A volume of 25–55 μl of solution (depending on immunotoxin concentration and size of the animal) was injected into the vitreous using a 30 gauge syringe needle. Prophylactic anti-inflammatory treatment included either IM injection of kenalog, 0.3 cc of 40 mg/ml, or oral 5 mg prednisone tablets. Additionally, the treated eye received a single dose of 0.3 cc kenalog, via subtenons injection, at the time of the procedure. Systemic anti inflammatories began 1 day prior to the procedure and continued for 1 week, or prn.

A subtenon injection of 1% atropine and 0.4 ml triamcinolone acetonide (Kenalog) was used to minimize inflammation. Additionally, animals 609, 527, 526, and 520 were pretreated with dexamethasone. The following measurements were recorded before and after treatment.

### Pupil testing

Pupillometry was performed ~1 month prior to injections, and 1–3 months after injections. For pupillography, animals were anesthetized with an intramuscular injection of 10 mg/kg ketamine and 1 mg/kg acepromazine, supplemented with a half dose approximately every 10 min. This minimal dose of ketamine was used to immobilize the animals while maintaining a similar heart rate as the awake state, minimizing sympathetic system suppression from anesthesia and maintaining a fully responsive pupil to light stimulation. Heart rate and blood oxygen were monitored with a pulse oximeter (model 9847V; Nonin Medical, Inc., Plymouth, MN, USA). The pupil of the right eye (the experimental eye) was dilated with 1% tropicamide. Animals were placed prone in a head holder and the lids were held open with an eyelid speculum. Custom made plano powered rigid gas permeable contact lenses were placed on each cornea with moisturizing lubricant (Refresh Celluvisc, Allergan) to maintain corneal integrity.

Stimuli were presented with a ColorBurst (Diagnosys, LLC, USA), positioned ~10 mm from the right eye and providing a 140° field of view. Long-wavelength stimuli were 651 nm (“Red”) with a half-max width of 25 nm and short-wavelength (“Blue”) stimuli were 456 nm with half-max width of 20 nm (Spectroradiometer CS1W, Minolta). Consensual pupil responses were recorded continuously in the left eye with an infrared eye tracker at 60 Hz (ViewPoint, Arrington, USA). The camera was focused at the pupil plane and calibrated at the beginning of each session.

**Figure 1:**
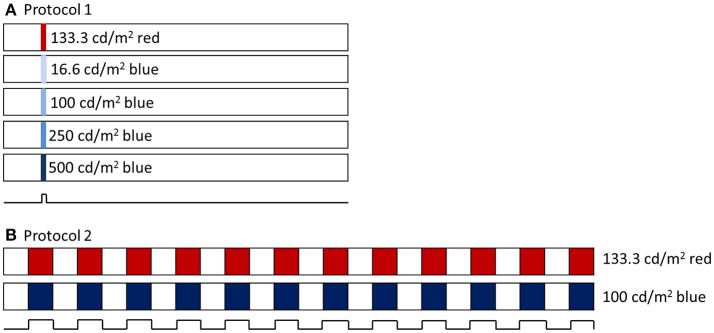
**(A)** Pupillometry protocol 1 included a 1 s long wavelength (red) stimulus and 4 increasing intensities of 1 s short wavelength (blue) stimuli. Stimuli were preceded with 10 s baseline and 60 s post illumination pupil recordings. **(B)** Pupillometry protocol 2 included a 2 min 0.1 Hz flickering long wavelength stimulus, followed by a 2 min 0.1 Hz flickering short wavelength stimulus.

Two experimental protocols were utilized (Figure 1). For both, baseline pupil diameter was first recorded for at least 10 s. For the first protocol, a 1 s long-wavelength 133 cd/m^2^ stimulus was presented (3.3 × 10^14^ photons/cm^2^/s), followed by four 1 s short-wavelength stimuli with increasing intensity, 16.6 cd/m^2^ (6.4 × 10^13^ photons/cm^2^/s), 100 cd/m^2^ (3.7 × 10^14^ photons/cm^2^/s), 250 cd/m^2^ (9.2 × 10^14^ photons/cm^2^/s), and 500 cd/m^2^ (1.5 × 10^15^ photons/cm^2^/s), with an interstimulus interval of at least 60 s. Following the first protocol, the second protocol was run, which included a 2 min 0.1 Hz flickering on and off long-wavelength 133 cd/m^2^ stimulus followed by a 2 min 0.1 Hz flickering on and off short-wavelength 100 cd/m^2^ stimulus.

Pupil data were analyzed offline in Excel (Microsoft Office 2013). Data were filtered to remove artifacts. For the first protocol utilizing 1 s stimuli, the following pupil metrics were quantified. Baseline pupil diameter was calculated as the average pupil diameter 10 s before the first stimulus. Peak pupil constriction, representing primarily a measure of rod/cone photoreceptor activity, was calculated for each stimulation as the smallest pupil diameter following light onset, relative to baseline pupil diameter. The post-illumination pupil response (PIPR) was quantified as the 6 s PIPR, which was calculated as the mean pupil diameter (relative to baseline) averaged over 6–7 s after stimulus offset. Paired *t*-tests were used to assess differences between baseline and follow up pupil constriction and 6 s PIPR.

For the second protocol utilizing 0.1 Hz flickering stimuli, data were normalized to the baseline as described above. Each 2 min period included twelve 5 s stimulus-on intervals. Peak pupil constriction during each of the 12 stimulus-on intervals, averaged for 0.5–4 s during each stimulus, for long-wavelength stimuli, was averaged. Pupil constriction was calculated in a similar manner for short-wavelength stimuli.

### Retinal imaging

Retinal structure was assessed with spectral domain optical coherence tomography (SD-OCT, Spectralis, Heidelberg, Germany) before and ~6 weeks and 6 months after immunotoxin injection. For imaging, the animals were anesthetized with an intramuscular injection of ketamine (15–20 mg/kg) and acepromazine (0.15–0.20 mg/kg). The animal's head was stabilized using a 5-way positioner (X, Y, Z, tip, and tilt) and gas permeable contact lenses were inserted to ensure optical clarity. The OCT's scan pattern (20° × 20°) was centered and focused on the fovea. Twenty horizontal scans were obtained using the instrument's highest resolution protocol resulting in B-scan images of 1,536 × 496 pixels; only scans with a quality index of 20 db or higher were analyzed. The instrument's auto re-scan feature was employed to track anatomic features to ensure that all subsequent scans were performed at the same retinal location as the baseline measurements. The SD-OCT instrument has an axial resolution of 3.9 μm per pixel (25). Scan data were exported and analyzed using custom Matlab software. An experienced observer manually segmented each scan to identify inner limiting membrane and retinal pigment epithelium. The center of the fovea was identified as the deepest point in the foveal pit observed in the central scans. Retinal thickness was averaged across the 6 mm line scan that passed through the fovea.

### Electrophysiology

ERG responses were recorded to assess potential effects of the immunotoxin on retinal function. For ERG recordings, animals were anesthetized intramuscularly with ketamine (20–25 mg/kg/hr) and xylazine (0.8–0.9 mg/kg/hr) and were treated with atropine sulfate (0.04 mg/kg injected subcutaneously), as previously described (26). Body temperature was maintained between 36.5 and 38°C with a thermostatically controlled blanket (TC1000 temperature controller; CWE, Ardmore, PA). Heart rate and blood oxygen were monitored with a pulse oximeter (model 9847V; Nonin Medical, Inc USA). Pupils were fully dilated to approximately 8.5 mm in diameter with topical tropicamide (1%) and phenylephrine (2.5%). ERGs were recorded using Dawson, Trick, Litzkow (DTL) electrodes (27) that were moistened with moisturizing lubricant (Refresh Celluvisc, Allergan) and positioned across the center of the cornea and under a corneal contact lens on each eye. A platinum wire inserted temporal to each eye served as the reference electrode, and a hypodermic needle in the skin of upper back as the ground electrode. Recordings were amplified and filtered (DC-300 Hz).

Full-field dark- and light-adapted ERG responses to brief flashes were recorded using an Espion3 system with a ColorDome stimulator (Diagnosys LLC, USA). Animals were dark-adapted for 30 min, and the scotopic stimuli were ISCEV standard white LED flashes of 0.01 and 3 cd s/m^2^ white LED flashes, and Xenon flashes of 10 and 100 cd s/m^2^, with three repetitions averaged (28). The photopic stimuli were brief red LED flashes (λ_max_ = 650 nm, 0.04–5.86 cd s/m^2^) on a rod-saturating blue background (λ_max_ = 462 nm, 10 photopic cd/m^2^), 10–20 repetitions averaged, to elicit responses from both outer and inner retina (26). ERGs were recorded before, soon after in some animals, and at 9 months after treatment in all animals, as indicated below.

ERGs were analyzed offline using a custom MATLAB program (MathWorks, Natick, MA). Amplitudes of dark- and light-adapted a-waves were measured from baseline to trough, and b-waves, from a-wave trough to b-wave peak, from records that were filtered, 0–75 Hz, to remove high frequency oscillatory potentials. Photopic negative responses (PhNRs) were measured from baseline to trough. For a-wave, b-wave, and PhNR, at baseline and 9 months after treatment, the percent difference in amplitude between the two eyes (OS-OD) was calculated using Equation (1):

(1) $$\begin{array}{l} Percent\;difference=-100^{*}\left(OS-OD\right)/OS \end{array}$$

### Immunohistochemistry

Animals were sacrificed ~6–9 months after injection. Both eyes of each animal were enucleated, globes were hemisected, vitreous removed, and the resulting eyecups were fixed in 4% paraformaldehyde for 1 h. The tissue was then rinsed and stored in 0.1 M phosphate-buffered saline (PBS), containing 0.3% sodium azide until processed for immunohistochemistry. Whole retinas were isolated from the eyecup and blocked in a blocking buffer containing 0.1 M PBS triton, 5% donkey normal serum, 3% monkey normal serum, and 0.3% sodium azide. The same blocking buffer was used for all primary antibody dilutions.

IpRGCs were double labeled with two different antibodies against melanopsin—one recognizing an epitope in a region of the C′ terminus, and one recognizing an epitope in the N′ terminus. The purpose of the use of the two labels for ipRGCs was two-fold. First, the double-label served as a specificity control. The OPN4 immunotoxin was conjugated to the N′ terminus antibody. IHC labeling with this antibody confirms the population of cells targeted for immunoablation. Second, both channels were assessed for fluorescence signaling to confirm ablation of the cells.

The retinas were labeled sequentially as previously described (29). Briefly, antibodies against C-terminal melanopsin (OPN4; diluted 1:10,000, 5 days at 4°C) were visualized using Envision (diluted 1:2, DAKO) and tyramide conjugated Alexa-594 (Molecular Probes). The retinas were then labeled with the same affinity-purified rabbit polyclonal antibodies against the OPN4 N-terminal antibodies that were used for toxin conjugation (diluted 1:1,500, 4 days at 4°C) and visualized with Alexa-488 donkey anti-rabbit (diluted 1:200; Jackson Immunoresearch Laboratories,). Cell nuclei were labeled with Hoechst nuclear stain (diluted 1:600).

Images were collected with a Nikon A1 Confocal microscope. Double-labeling with both antibodies was confirmed using single optical sections. Counts of OPN4-immunoreactive and Hoechst-labeled cells were obtained from 4 regions of interest (ROI) taken in central retina (~1 mm superior, inferior, nasal of the macula, and ~1 mm temporal of the ONH), and 4 ROIs taken at >3 mm superior, inferior, and temporal of the macula and >3 mm nasal of the ONH (mid-peripheral/peripheral), and averaged for each retina. The counts were obtained from confocal image stacks and reflect both inner and outer populations of OPN4-immunoreactive ganglion cells. Labeling patterns were confirmed in stitched confocal images of the entire retina. Because there were no significant differences in the number of OPN4-immunoreactive cells in the control eyes, the control retina counts were pooled. Image J was used to perform semi-automated cell counts (30) and adjust brightness and contrast. GraphPad prism was used for statistical analysis. Figures were finalized using Adobe Photoshop.

## Results

Following immunotoxin injection, all eyes demonstrated some ocular inflammation that ranged from very mild (for the lowest dose) to more severe (for the highest dose). The control eye for one animal, 526, experienced inflammation that led to media opacities; therefore, post-injection pupillography, electrophysiology, and imaging could not be performed for this animal. Post-operative inflammation was treated with systemic dexamethasone.

### Pupillography

**Figure 2:**
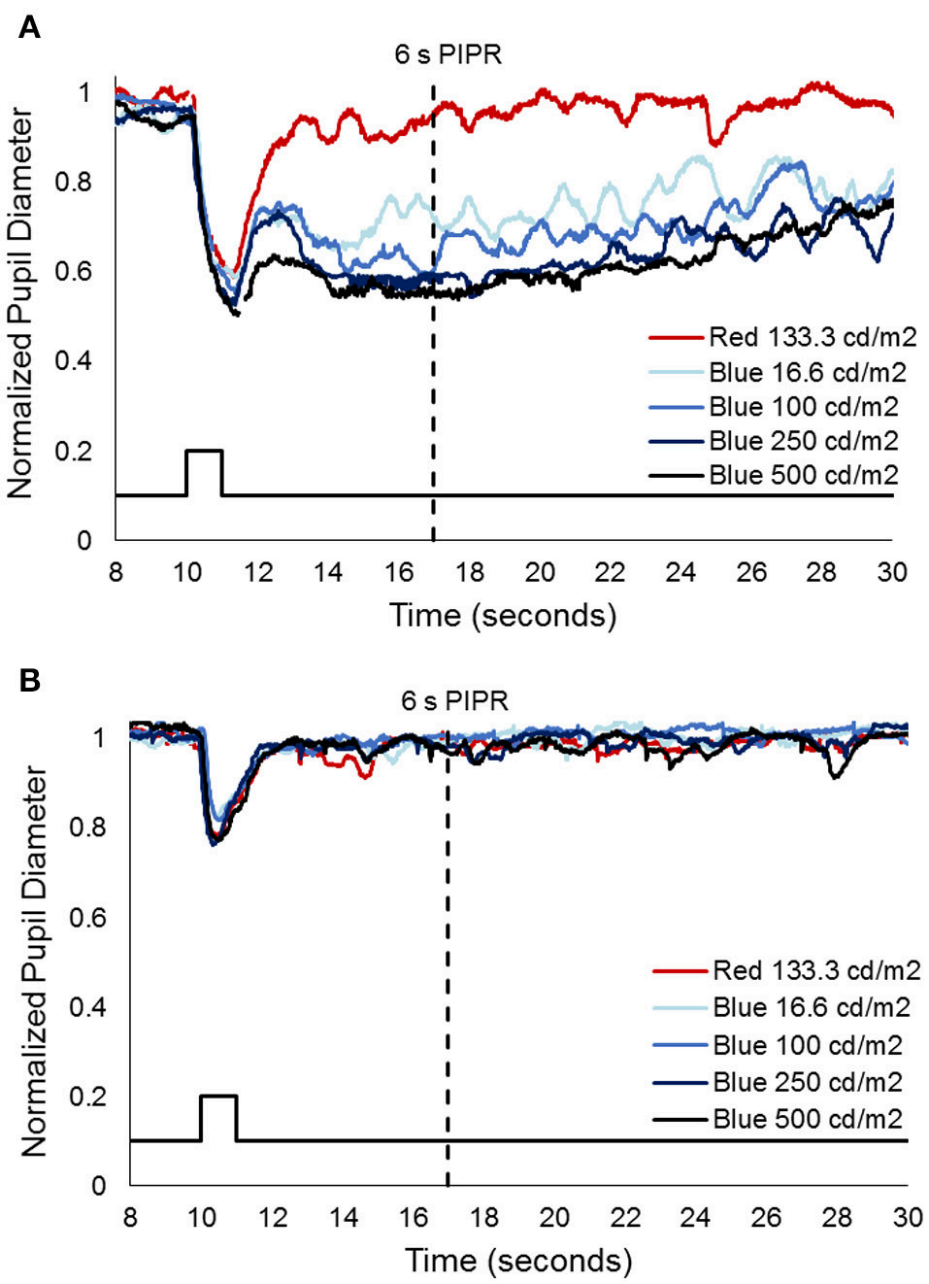
Representative pupil measurements from one animal, 552, **(A)** before and **(B)** after immunotoxin injection (10 μg). Stimuli included 1 s long wavelength (red) and 4 increasing intensities of short wavelength (blue) pulses. Solid black line represents the stimulus and the dashed black line represents the 6 s post-illumination pupil response (PIPR).

Prior to injection, pupil responses were similar for all animals. Smaller values for constricted pupil size and the PIPR indicate a smaller pupil diameter, i.e., stronger pupil light response. Before treatment, animals showed robust rod/cone and melanopsin-driven pupil responses. For the first protocol (Figure 2), 1 s long-wavelength (Red) stimuli resulted in an initial pupil constriction that reached 0.66 ± 0.08 of baseline, then rapidly redilated following light offset with a 6 s PIPR of 1.0 ± 0.05, indicating that the pupil had returned to baseline by 6 s after light offset (Tables 2, 3). For increasing intensities of short-wavelength (Blue) stimulation, initial pupil constriction was 0.67 ± 0.08 (lowest intensity, 16 cd/m^2^) to 0.60 ± 0.07 (highest intensity, 500 cd/m^2^). The responses were characterized by a rapid pupil constriction, followed by rapid partial redilation at light offset, with subsequent reconstriction. The reconstriction amplitude demonstrated a graded response, with the greatest reconstriction for the highest intensity short-wavelength stimuli. The pupil gradually redilated back to baseline over the following 10–60 s. The 6 s PIPR for the lowest intensity stimulus was 0.81 ± 0.09 and for the highest intensity stimulus was 0.70 ± 0.12, indicating slower redilation for the highest intensity.

**Table 2:** Normalized constricted pupil size to 1 s long wavelength (red) and 4 increasing intensities of short wavelength (blue) stimuli before and after immunotoxin injection.

**Subject ID**	**Dose (μg)**	**Red 133 cd/m**^**2**^		**Blue 16 cd/m**^**2**^		**Blue 100 cd/m**^**2**^		**Blue 250 cd/m**^**2**^		**Blue 500 cd/m**^**2**^	
		**Before**	**After**	**Before**	**After**	**Before**	**After**	**Before**	**After**	**Before**	**After**
550	50	0.67	0.91	0.67	0.86	0.69	0.97	0.66	0.96	0.66	0.83
552	10	0.56	0.82	0.59	0.86	0.55	0.85	0.53	0.83	0.5	0.8
520	10	0.64	0.75	0.63	0.79	0.6	0.8	0.6	0.74	0.57	0.77
527	1	0.78	0.94	0.8	0.94	0.71	0.91	0.67	0.88	0.66	0.87
609	0.316	0.65	0.89	0.66	0.89	0.61	0.84	0.61	0.78	0.6	0.74

**Table 3:** 6 s PIPR to 1 s long wavelength (red) and 4 increasing intensities of short wavelength (blue) stimuli before and after immunotoxin injection.

**Subject ID**	**Dose (μg)**	**Red 133 cd/m**^**2**^		**Blue 16 cd/m**^**2**^		**Blue 100 cd/m**^**2**^		**Blue 250 cd/m**^**2**^		**Blue 500 cd/m**^**2**^	
		**Before**	**After**	**Before**	**After**	**Before**	**After**	**Before**	**After**	**Before**	**After**
550	50	0.96	1.01	0.8	1.01	0.78	1.05	0.79	1.05	0.74	1.05
552	10	0.96	0.98	0.72	1	0.67	1	0.59	0.97	0.55	0.97
520	10	0.98	0.97	0.76	1	0.68	1	0.64	1.02	0.63	1
527	1	0.98	1.01	0.96	0.99	0.93	0.94	0.91	0.98	0.87	0.96
609	0.316	1.1	1	0.83	0.97	0.75	0.94	0.71	0.92	0.72	0.88

After injection, pupil responses to 1 s long-wavelength and all intensities of short-wavelength stimuli decreased for all concentrations of immunotoxin tested, with the lowest immunotoxin concentration (0.316 μg) generally having the least effects. The initial pupil constriction to both long- and short-wavelength stimuli significantly decreased (i.e., less constriction/larger pupil, *p* < 0.002 for all). Additionally, the PIPR to short-wavelength stimuli significantly decreased, being completely eliminated for all but the highest intensity stimulus (*p* < 0.02). The 6 s PIPR was not significantly different for long-wavelength stimuli before and after the immunotoxin; in all cases, the 6 s PIPR was >0.96.

**Figure 3:**
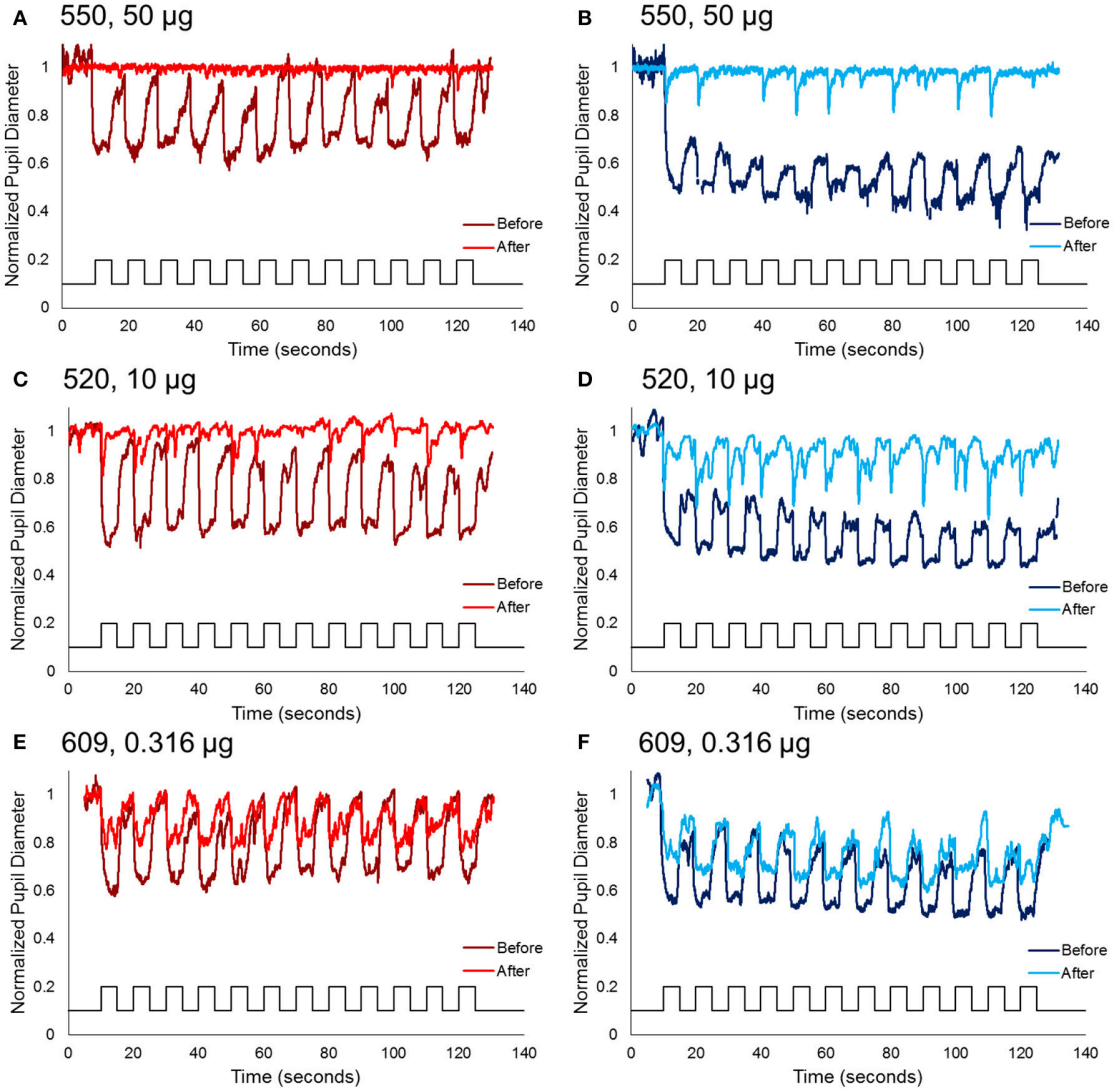
Normalized pupil diameter for right eyes to a 2 min 0.1 Hz long wavelength (left column) or short wavelength (right column) stimuli. Dark red and blue traces represent the pupil before immunotoxin injection, and light red and blue traces represent the pupil 1–3 months after injection. **(A,B)** Animal 550, receiving the highest concentration immunotoxin (50 μg), **(C,D)** animal 520, receiving a midrange concentration immunotoxin (10 μg), and **(E,F)** animal 609, receiving the lowest concentration immunotoxin (0.316 μg).

For the second protocol, using 0.1 Hz flickering on and off long-wavelength stimuli for 2 min, followed by short-wavelength stimuli for 2 min, responses prior to injection showed rapid pupil constriction at each stimulus onset that was maintained for the duration of the 5 s stimulus-on interval across 12 stimuli (Figure 3). In general, for long-wavelength stimuli, the pupil constriction during stimulus-on demonstrated a slight attenuation over the 2 min period, whereas, for short-wavelength stimuli, the pupil constriction during stimulus-on demonstrated slight potentiation over the 2 min period. On average across the 12 stimuli and for all subjects, constricted pupil size for long-wavelength stimuli was 0.68 ± 0.1, and for short-wavelength stimuli was 0.49 ± 0.02.

Following immunotoxin injection, pupil responses to 0.1 Hz long- and short-wavelength stimuli significantly decreased, and were essentially eliminated for the highest immunotoxin concentrations. For all concentrations of immunotoxin, constricted pupil size to long-wavelength stimuli was 0.9–1.0. Constricted pupil size to short-wavelength stimuli was 0.84–0.96. Of importance, for all immunotoxin concentrations except for the lowest dose, pupil constriction, if present at all, was transient and did not persist for the 5 s stimulus duration.

### Retinal imaging

**Figure 4:**
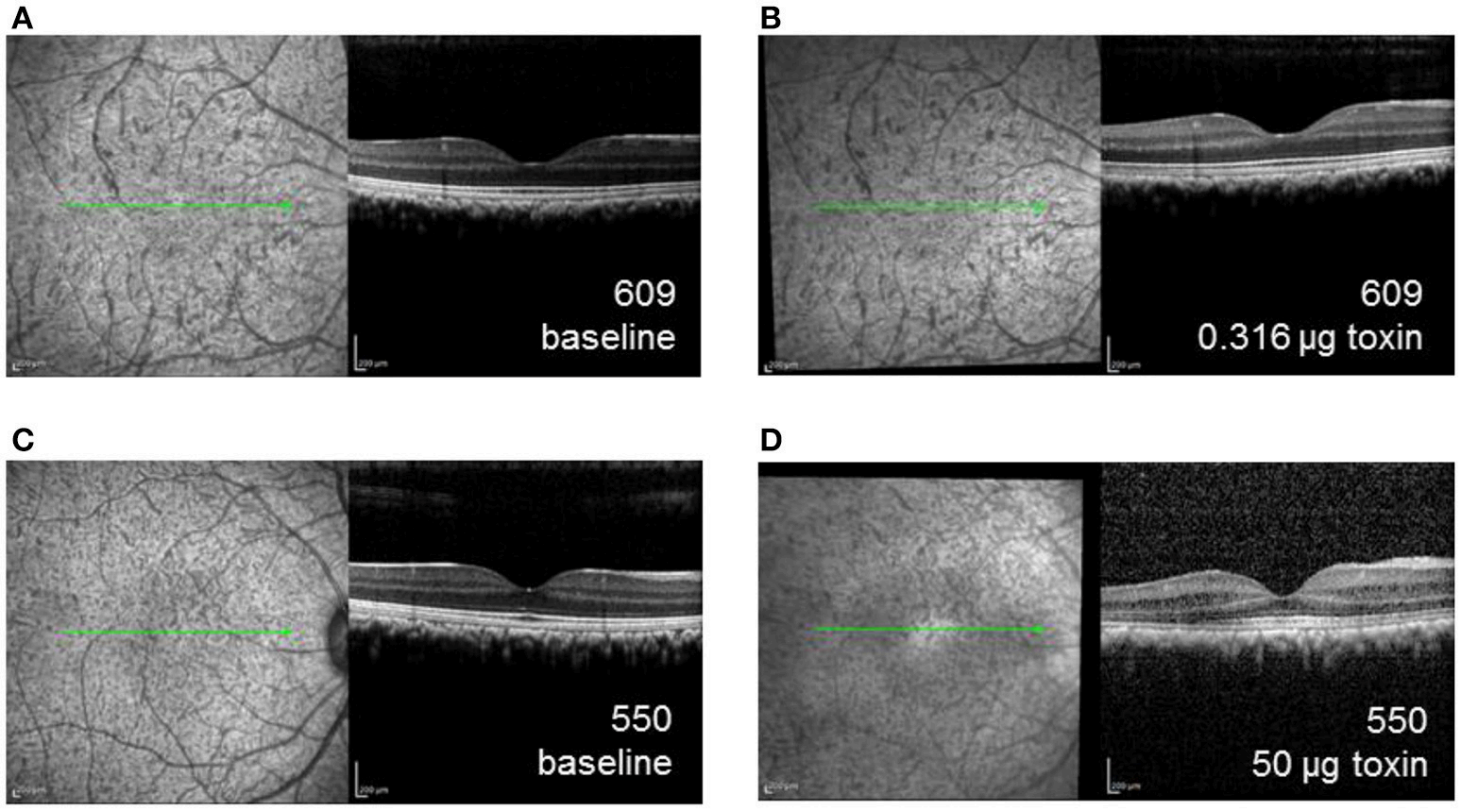
SD-OCT images of the right eye for animal 609, which received the lowest dose immunotoxin (0.316 μg), **(A)** prior to injection and **(B)** 6 weeks after injection demonstrating well-preserved retinal structure, and for animal 550, received the highest dose immunotoxin (50 μg), **(C)** prior to injection, and **(D)** 6 weeks after injection, demonstrating some disruptions of normal retinal layers.

Retinal thicknesses of control eyes of were not significantly different before and after the injection for all the monkeys (*p* = 0.32). For experimental eyes, dose-dependent inflammation and structural changes were observed in posterior segment SD-OCT images. For example, the experimental eye of the monkey receiving the lowest dose (609-OD, 0.316 μg) did not exhibit structural changes or retinal thickness differences after 6 weeks or after 6 months of injection compared to baseline (Figures 4A,B). For 609-OD, baseline total retina thickness across the macular region was 296.8 ± 4.02 μm. At 6 weeks after injection, total retina thickness was 304.8 ± 23.22 μm, and at 6 months after injection was 298.25 ± 26.8 μm. However, the experimental eye of monkey 520 (10 μg) showed retinal thinning of 10 μm and of monkey 527 (10 μg) demonstrated retinal thickening of 45 μm. These changes likely represent general retinal inflammation rather than specific ablation of ipRGCs. The retinal thickness of the animal receiving one of the highest does (552, 25 μg) could not be obtained following injections due to poor optical quality resulting from inflammation. In the animal receiving the highest dose, 550 (50 μg), small opacities in the vitreous space representing inflammatory cells and fibrous membranes were observed with a slit lamp biomicroscope and were evident with retinal imaging after injection (Figures 4C,D).

### Electrophysiology

**Figure 5:**
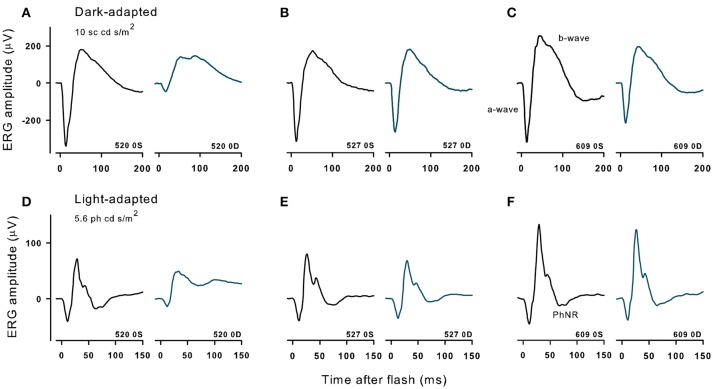
Dark- and light-adapted ERGs in control eyes (OS) and in experimental eyes (OD) of three different animals with high (520, 10 μg, **A,D**), midrange (527,1 μg, **B,E**) and lowest (609, 0.316 μg, **C,F**) doses of immunotoxin recorded 9 months after treatment. Responses are shown to 10 cd s/m^2^ bright flashes from darkness (top) and 5.86 cd s/m^2^ red flashes on a rod saturating blue background.

Figure 5 shows dark-adapted responses to a 10 cd s/m^2^ flash (top) and light-adapted ERG responses to a 5.6 cd s/m^2^ flash (bottom) recorded in three monkeys that received different doses of the immunotoxin 9 months prior. The immunotoxin reduced ERG amplitudes in the injected (right) eye substantially for the higher doses. However, animals receiving lower doses had better preserved outer retinal function, measured by a-wave (photoreceptor) and b-waves (bipolar cells), and inner retinal function measured by the PhNR (retinal ganglion cells) (31) in the injected eye than animals receiving higher doses. Prior to injection, at baseline, ERG amplitudes in the two eyes of each animal were generally similar (not shown) to the control (left) eye records shown in Figure 5. Baseline amplitudes were greater in the left eye in some animals, and the right eye in others, but the amplitude difference in stable recordings was within about 30% for each of the waves of the dark- and light-adapted ERG.

For the two lowest doses of the immunotoxin, percent differences in ERG amplitudes between the two eyes hardly exceeded differences seen in baseline recordings. For the dose of 0.316 μg (Figure 5C), a- and b-waves of dark- and light-adapted ERG of injected right eye had slightly lower amplitudes, but the differences were within 32% of the amplitude of the left eye, and the PhNR was the same in both eyes. For a higher dose of 1 μg (Figure 5B), ERG amplitudes for the injected eye again were slightly lower, but with 20% of the amplitude for the left eye except for PhNR amplitude, which was within 40%. In contrast, for the higher dose, 10 μg (Figure 5A), amplitudes were greatly reduced for all waves of the ERG with differences of 65% or greater for dark-adapted a- and b-waves, and the light-adapted a-wave. For the light adapted b-wave the difference was 47% and for the PhNR, which was no longer a negative wave, there was 205% difference from the left eye. In another monkey that received the 10 μg dose (not shown), dark and light-adapted ERG amplitudes also were greatly reduced.

### Immunohistochemistry

**Figure 6:**
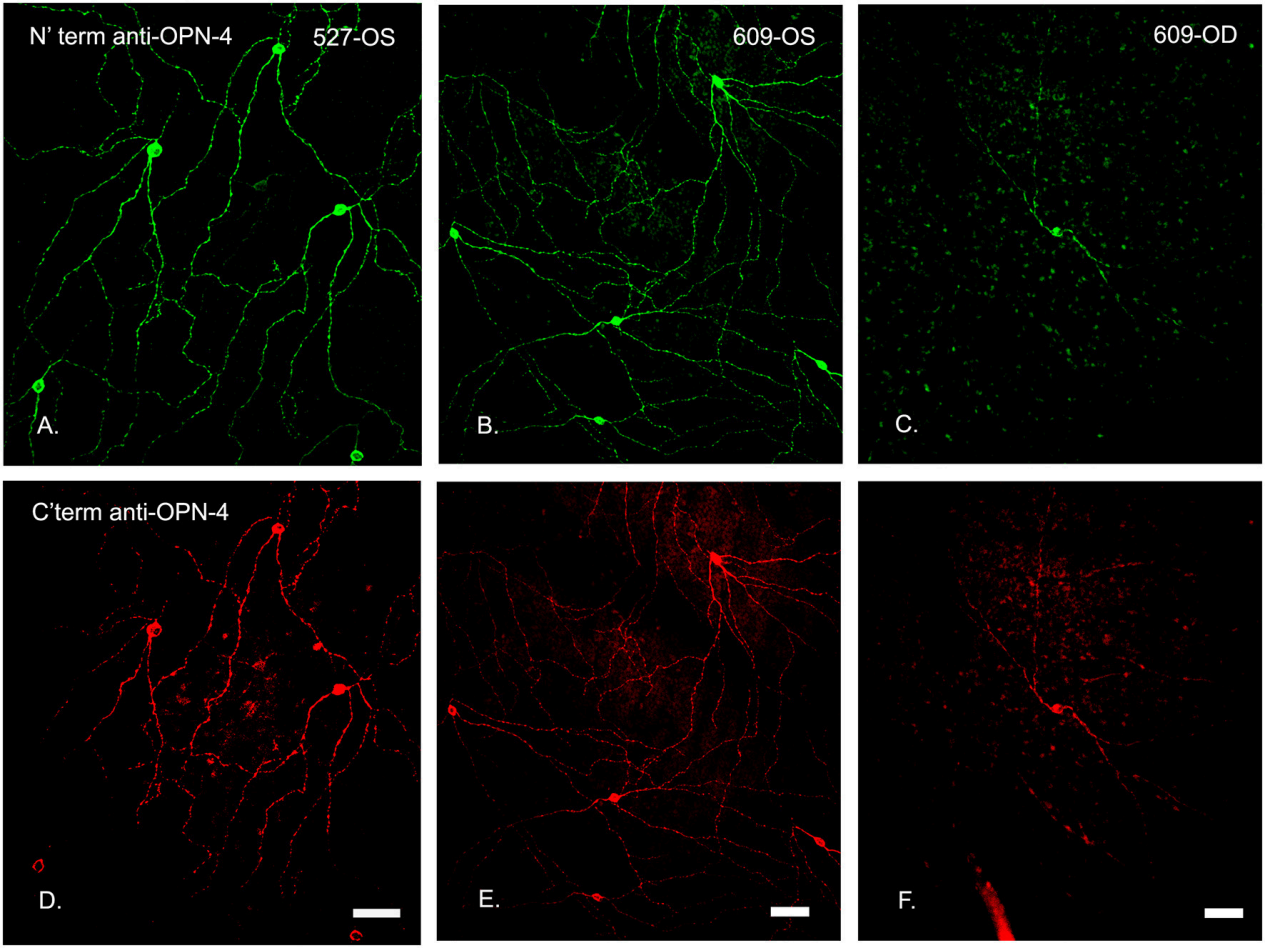
N′ and C′ terminal antibody IR was colocalized in the same cell populations in an untreated control eye. In central temporal, non-macular, retina, N′ and C′ terminal antibody IR **(A–F**, respectively) was colocalized in the same cell populations for both the control (OS, left and middle panels) and treated (OD, 0.316 μg, right panels) retinas, shown for animal 609. Note that the only labeled cell in this region of the treated retina had fewer branches. Scale bars for all panels = 50 μm.

Specificity for the OPN4 antibody in a control eye is shown in Figure 6. Immunohistochemistry showed that both N′ terminus and C′ terminus antibodies labeled the same populations of ipRGCs under all conditions, confirming that the population of cells targeted for immunoablation were melanopsin containing ganglion cells. Loss of fluorescent signaling on both channels confirmed complete ablation of the cells. The loss of processes on the few remaining ipRGCs suggests that the immunotoxin affected the health of those cells that were not ablated.

**Figure 7:**
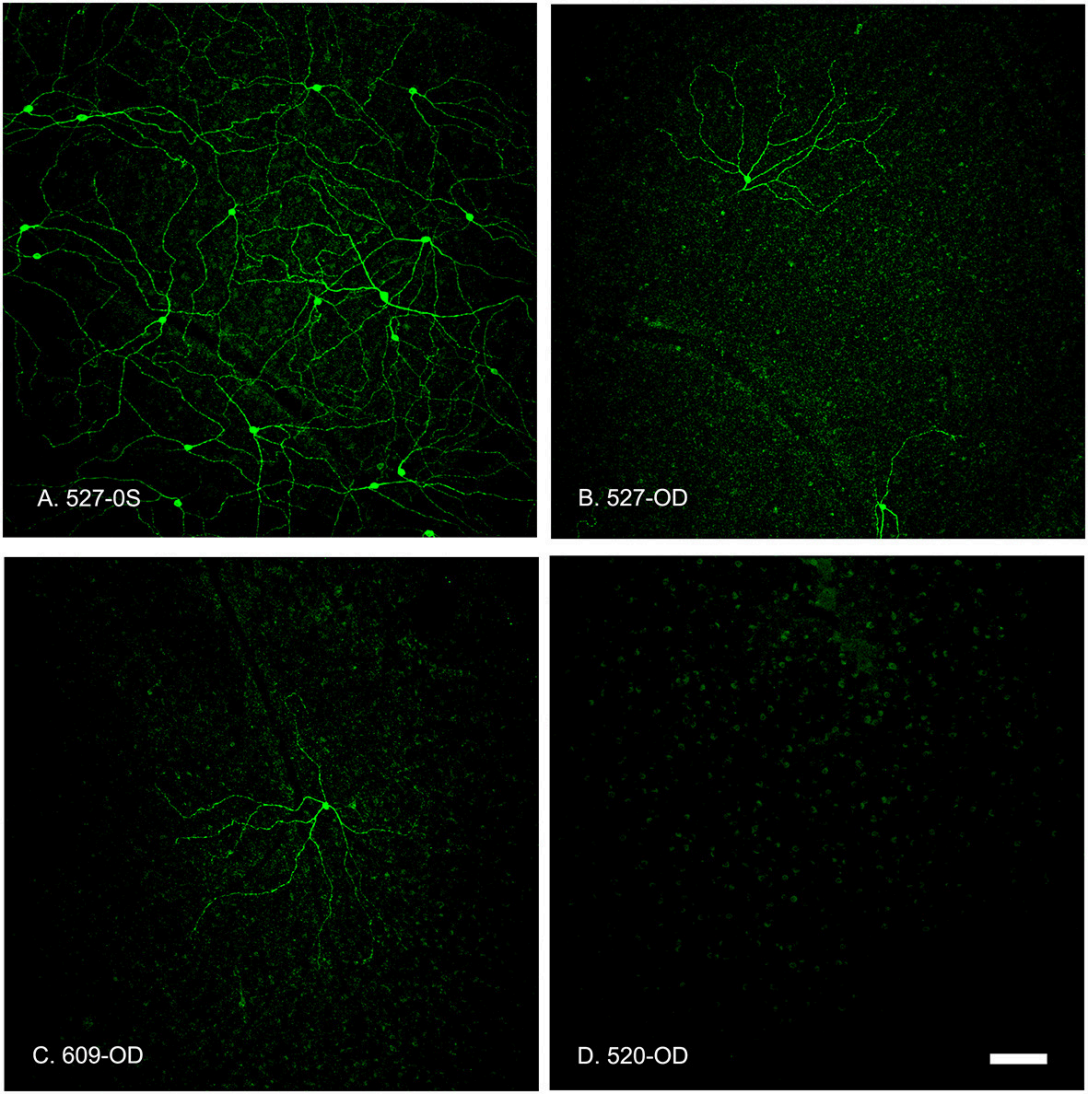
Summed z-stack projections of mid-peripheral retina taken at 10x magnification showed that the OPN4-IR cells provided coverage of the entire retinal section in control conditions (**A**, 527-OS). Retinas treated with increasing concentrations of OPN4-Saporin had few or no OPN4-IR cells. **(B)** 527-OD: 1 μg; **(C)** 609-OD: 0.316 μg; **(D)** 520-OD: 10 μg; Scale bar = 100 μm.

**Figure 8:**
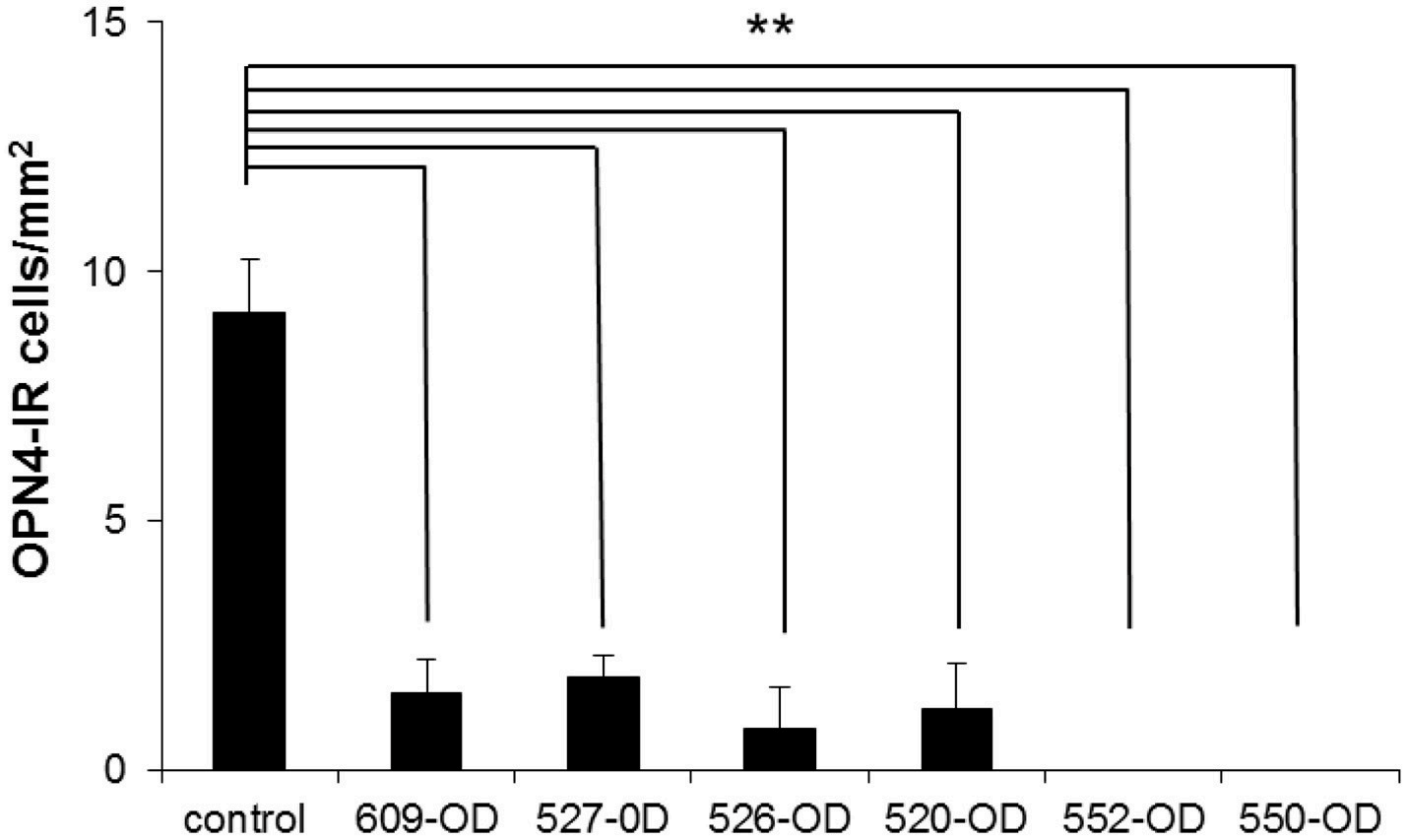
The number of OP4-immunoreactive cells in 4 regions of interest (ROI) taken in central retina (~1 mm superior, inferior, nasal, and temporal of the ONH) and 4 ROIs taken at >3 mm superior, inferior, nasal, and temporal of the ONH (mid-peripheral/peripheral) were averaged for each retina. Because there were no significant differences in the number of OPN-4 labeled cells in the control eyes, the control count was averaged from both retinas of one untreated control animal and the control eyes of the treated animals (*n* = 8 retinas). Because there were no systematic patterns regions of spared cells in the treated eyes, central, mid-peripheral, and peripheral ROIs in each animal were averaged. Labeling in the treated eyes was significantly reduced as compared to control (^**^P < 0.001, cells/mm^2^ + SEM). 609-OD: 0.316 μg; 527-OD: 1 μg; 526-OD: 3.16 μg; 520-OD, and 552-OD: 10 μg; 550-OD: 50 μg.

The number of OPN4-immunoreactive cells in control eyes, averaged from 8 regions of interest, was 9.2 ± 1 cells/mm^2^. In experimental eyes, there was an 80–100% reduction in the number of labeled ipRGC cells as compared to contralateral control eyes (*p* < 0.001, Figures 6–8). There were no labeled iPRGC cells in the eyes the received the two highest doses of toxin, and 1.87 (80% reduction) to 1.56 (83% reduction) cells per mm^2^ at the lower toxin concentrations. However, with the highest toxin concentrations, some ipRGC loss could result from inflammation, as evidenced by a trend toward reduced numbers of Hoechst labeled cells in these retinas. The overall ANOVA was significant at *p* = 0.05, although *post-hoc* pairwise comparisons were not.

## Discussion

The goal of this study was to develop and validate a targeted immunotoxin for *in vivo* ablation of ipRGCs in primates. Immunostaining showed that ipRGCs were targeted, and structural and functional evaluation suggested that other retinal cells were generally spared. Prior studies using OPN4-saporin conjugates had reported effective doses of 400 ng in the mouse eye (21) and 950 ng in the rat eye (22). The volume of the vitreous humor of the mouse eye is approximately 5 μl while that of the rat eye is ~40 μl, resulting in effective vitreal concentrations of 80 μg/ml for mouse and 24 μg/ml for rat. The vitreous humor volume of the Rhesus monkey is approximately 2 ml. We therefore extrapolated from rat to Rhesus monkey to establish our highest dose as 50 μg per globe. With the higher doses of immunotoxin (≥10 μg), 100% of the ipRGC population appeared depleted based on immunohistochemistry. However, these higher doses also resulted in intraocular inflammation that reduced outer and inner retinal function as measured by ERG. The inflammation observed at these higher immunotoxin doses is most likely a response to either the affinity-purified rabbit polyclonal antibody or to the saporin conjugate. Humanized antibodies can be administered at doses of 1.5 mg to macaque monkeys without any inflammatory response (32), so it is possible that the use of a less immunogenic antibodies (e.g., murine, camelid, nanobodies) or Fab fragments might reduce the observed inflammatory responses at higher immunotoxin doses.

While there was not a clear dose-response curve, the two highest doses resulted in complete loss of ipRGCs, and the four lower doses resulted in ~80% cell loss. It is possible that different degrees of functionality for the remaining cells existed, in that cells can lose function while still present. It is important to note that the lower 2 doses of immunotoxin, 0.316 and 1 μg, were sufficient to reduce the ipRGC population to ~20% of control, and significantly decreased the pupillary light reflex, with minimal inflammation that resolved within the post-injection period, and minimal impact on ERG measures of retinal function. It is likely that a more aggressive pre/post injection steroid regimen would allow for these and somewhat higher doses to be used with minimal post-injection inflammation. Further, at these two lower doses, the post-illumination pupil response was eliminated, suggesting that melanopsin-containing ipRGCs were no longer contributing significantly to pupillary responses or potentially to circadian responses. This would be consistent with studies in mice that showed that an immunotoxin induced decrease of 57% of ipRGCs was sufficient to induce significant changes in circadian behaviors (22).

The normal primate pupil has been shown to constrict rapidly to a light stimulus then, in some cases, exhibit a brief dilation at light cessation, followed by sustained reconstruction (33, 34). This pupil light reflex pattern was demonstrated in the present study in normal eyes (i.e., prior to immunotoxin injection) using short wavelength stimuli. After ablation, a transient and decreased pupil constriction was observed that rapidly returned to baseline, with no apparent sustained reconstriction that is the signature of melanopsin-driven pupil input.

Following injection of the immunotoxin, pupil responses decreased in both the amplitude of pupil constriction and in duration of the response. In primates, ipRGCs provide the major retinal input to the pretectal olivary nucleus (35), the control center for pupil control. Following ipRGC ablation, the pupil response to both long wavelength and short wavelength stimuli was significantly reduced, however, some residual pupil response was observed. These findings are consistent with the ipRGCs being the major conduit from the retina for both cone, and melanopsin-driven control of the pupil. This is likely the pathway for rod mediated pupil control as well; however, rod contributions were not specifically tested here. It is possible that some other non-melanopsin RGCs may also project to the pretectum in primates, and these may drive the residual pupillary responses that were observed. Alternatively, the residual pupil response may have been driven by the few ipRGCs that remained after abalation with the lower immunotoxin concentrations.

Studies in mice with genetic ablation of the ipRGCs demonstrated that the ipRGCs are the primary conduits for rod and cone input to non-image-forming visual responses (36, 37). These findings were confirmed in rodents using immunotoxin-induced ablation (23). While genetic and immunotoxin studies have been performed in rodents to clarify the role of ipRGCs, no studies to date have blocked ipRGC transmission in the presence of functioning outer retinal pathways in primates.

Further refinement and validation of this melanopsin-targeted immunotoxin and experiments utilizing binocular ablation will allow for better clarification of the functions of ipRGCs in primates. Future studies that include immunohistochemical testing of other retinal cell types, including rods, cones, bipolar, amacrine, and other types of ganglion cells will provide information on absolute specificity. An application of ipRGC ablation in macaque monkeys would be to investigate the role of ipRGCs in light-mediated behaviors such as circadian entrainment, phase shifting, and masking. Another potential application of immunoablation includes ipRGC depletion in infancy, during the emmetropization process, to understand potential roles of ipRGC transmission in eye growth and refractive error development. Recent studies in mice suggest that an interaction between refractive development and circadian biology exists (38). Mice lacking melanopsin have abnormal refractive development and increased susceptibility to form-deprivation myopia. However, melanopsin contributions to eye growth are not yet understood. IpRGCs have been shown to communicate with axon terminals of dopaminergic amacrine cells (8). Early studies in human retinae suggest that synapses transmit information from amacrine cells to ipRGCs (39), whereas recent investigations in mouse and macaque retinae suggest that ipRGCs transmit information to amacrine cells (40). Dopamine is a known neuromodulator in refractive development (41). Retinal dopamine is reduced in form deprivation myopia (42). Additionally, administration of dopamine agonists reduced myopia development in animal models (43, 44). Observed relationships between dopaminergic amacrine cells and ipRGCs have led to speculation that melanopsin driven activity may play a role in eye growth and myopia.

In summary, our findings show that a newly developed OPN4-saporin immunotoxin, at concentrations that preserve retinal structure and function, is highly specific for primate ipRGCs. An optimal concentration that maximized ipRGC elimination with minimal inflammation that would be appropriate for future studies is on the order of 0.5–1 μg, which can be used to produce a substantial reduction in ipRGC numbers, as well as effectively eliminating melanopsin-driven pupil responses.

## Author contributions

LO, CS, PG, LF, ES, L-FH, and BA: manuscript preparation. KC: immunotoxin development. LO and AJ: pupil experiments. LF: electroretingrams. L-FH and BA: OCT imaging, post treatment care. CS: histology.

### Conflict of interest statement

The authors declare that the research was conducted in the absence of any commercial or financial relationships that could be construed as a potential conflict of interest.
